# Impact of virtual group-based acceptance and commitment therapy on social adjustment and work-family conflict among intern nurses: a randomized control trial

**DOI:** 10.1186/s12888-023-05045-8

**Published:** 2023-08-01

**Authors:** Ayman Mohamed El-Ashry, Eman Sameh Abd Elhay, Samah Mohamed Taha, El Saied Abd El Hamid El Sayed Salem, Mona Metwally El-Sayed

**Affiliations:** 1 Psychiatric and Mental Health Nursing, Faculty of Nursing, Alexandria University, Alexandria, Egypt; 2 Psychiatric and Mental Health Nursing, Faculty of Nursing, Mansoura University, Mansoura, Egypt; 3 Fitness, Gymnastics and Sports Show, Faculty of Physical Education for Men, Alexandria University, Alexandria, Egypt

**Keywords:** Acceptance and commitment therapy, Intern nurses, Social adjustment, And work-family conflict

## Abstract

**Background:**

An action-oriented approach such as acceptance and commitment therapy may help reduce the fusion of conflicting ideas, empower new intern nurses to act according to their values, and maximize their psychological flexibility.

**Objective:**

To evaluate the impact of a virtual group-based acceptance and commitment therapy intervention on intern nurses’ social adjustment and work-family conflict.

**Design:**

A parallel, single-blind randomized control trial on intern nurses (*n* = 70) was randomly allocated to either a six-session online acceptance or commitment therapy intervention (*n* = 35) or a waiting list control group (*n* = 35), with each session lasting 90 min.

**Measures:**

The work-related acceptance and action questionnaire, the social adjustment scale-self report, and the work-family conflict scale before, after, and one month after the intervention.

**Results:**

The psychological flexibility mean score of the study group was significantly higher than that of the control group (43.11 vs. 34.15, *p* < .001) immediately after the intervention, and this effect was sustained one month after the intervention (41.88 vs. 33.21, *p* < .001) with a more significant effect size (F = 128.457, *p* < .001, η2 = 0.791). The social adjustment mean score of the study group had significantly improved in all four subscales, with statistically significant differences (*p* < .001). One month after the intervention, the study group had significantly higher scores than the control group in total score, with statistically significant differences (*p* < .001) and large effect sizes (η2 = 0.932). Work-family conflict mean score of the study group was decreased immediately after the intervention, with statistically significant differences (*p* < .001). One month after the intervention, the study group had significantly lower scores than the control group in all three subscales of the WFCS, with statistically significant differences (*p* < .001) and large effect sizes (η2 = 0.943).

**Conclusion:**

Our findings proved that the virtual group-based ACT intervention effectively improved psychological flexibility and social adjustment, reducing work-family conflict among intern nurses. These findings suggest that the virtual group-based ACT intervention can be a practical approach to improving intern nurses’ mental health and well-being, which could affect their job performance and overall quality of life.

**Trial registration:**

The study was registered retrospectively as a randomized clinical trial on 10/2/2023, reference number; NCT05721339.

## Introduction

An internship is an opportunity for professional development that gives students real-world experience in a field linked to their studies or desired career [1]. It can be challenging to go from being a student to a professional stage in life [2]. It is generally known that most intern nurses experience difficulties in their daily work [3].

One of the most challenging issues for intern nurses is work-family conflict (WFC). Due to the demanding nature of their profession, this entails lengthy shifts, a great deal of emotional and physical stress, and intense patient involvement [4]. According to Greenhaus and Beutell [5], a work-family conflict arises when one role (work or personal life) imposes obligations and demands that are incompatible with the other position. Work-family conflict has a negative impact on the overall healthcare system, increasing absenteeism and turnover while decreasing nurses’ productivity and performance in terms of the quality of patient care provided [6]. Ever since, WFC has been associated with lower levels of life satisfaction and quality of life. Studies have found a direct correlation between psychological conflict, destructive behaviors, and depression [7, 8].

Social adjustment is getting along with other people in a group or community. Numerous studies have found a positive correlation between social adjustment and achievement motivation. Mirmoeini, Bayazi, & Khalatbari [9] found that people with low social adjustment sometimes struggle with verbal communication with others, making plans, caring for themselves, and carrying out everyday tasks. From another perspective, those new nurses need to adjust to people from different cultures, and the new environment is one of the most crucial parts of their experience [10]. Therefore, nurses with high levels of social adjustment will also have high levels of achievement desire [11, 12].

To this end, researchers have adopted virtual mindfulness-based workplace training programs to avoid and minimize intern nurses’ conflict [13]. Such therapy may be simple, cost-efficient, and rapidly disseminated to the nursing population. This novel therapy approach may reduce the need for counseling services and increase the number of individuals treated. Virtual group-based learning may also improve access for remote students [14]. Acceptance and commitment therapy (ACT) is an illustration of these approaches. ACT is being developed with healthcare workers [15]. ACT belongs to a group of third-wave or contextual cognitive-behavioral therapy approaches that focus on altering or changing the relationship of individuals with their unhelpful thoughts and feelings rather than directly trying to change the form or frequency of these internal experiences [15]. Additionally, studies have shown that ACT can enhance other employees’ well-being and performance indicators, such as fostering workplace innovation [16].

The ACT model is conceptually based on the behavioral analytic account of language called Relational Frame Theory (RFT), which aims to reduce the influence of thoughts and language to increase the number of choices concerning following a valued life path [17]. Alternatively, with another meaning, the ultimate goal of ACT is to “bring verbal cognitive processes under better contextual control and encourage the intern nurse to spend more time in touch with the positive effects of his or her actions as part of a valued life path immediately in the present moment [18]. In this respect, Mokhtari, Rezaee, & Baljani [19] confirmed the relationship between work-family conflict and social adjustment among hospital clinical personnel.

**Fig. 1:**
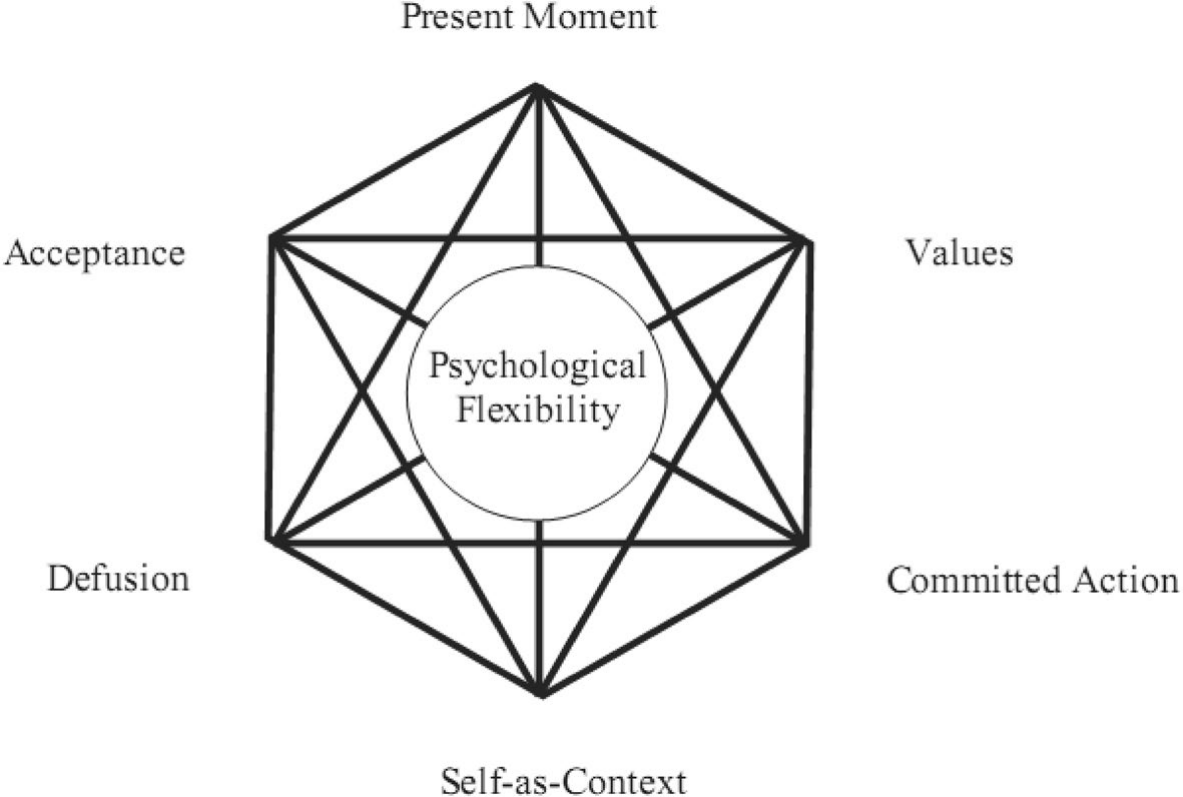
The ACT model of psychological flexibility, or HEXAFLEX [18]

Balancing work and family life is challenging and conflicting for any adult needing more psychological flexibility. Sabil et al. [20] verified that when people begin to work, they struggle to balance jobs and family life. Combining work and family is beneficial. The HEXAFLEX model in Fig. 1 demonstrates ACT’s six main theoretical processes that work together to improve psychological flexibility. Although the six processes in the model are described as separate, they are highly interdependent, so beginning to use one strategy is likely to affect the others positively. Subsequently, the absence of one or more of the six psychological flexibility strategies will lead to a risk of psychological inflexibility, which is believed to be the root cause of human suffering and conflicts [18].

Overall, several studies have applied ACT therapy, and the results indicate the effectiveness of this approach in reducing job stress, behavioral and psychosocial problems [21–23], as well as increasing the well-being, quality of life, resilience, and social and health adjustment of nurses [24, 25].

### Significance of the study

On the other hand, a preliminary study of ACT interventions regarding such variables as social adjustment and work-family conflict among intern nurses is a research gap that will probably be filled with this study. As well as the results of this study will have a significant impact on the profession of nursing. Intern nurses struggle with social adjustment and work-family conflict, and resolving these problems is essential for enhancing their well-being, work satisfaction, and patient care. For reducing work-family conflict and enhancing social adjustment among intern nurses, the adoption of virtual mindfulness-based workplace training programs, such as ACT intervention, has the potential to be a straightforward, affordable, and readily shared strategy. If this study is successful, it may show that ACT intervention can enhance nursing interns’ psychological and social well-being. As a result, nursing education and training programs may incorporate these interventions to promote intern nurses’ mental health and general well-being.

Therefore, the primary outcome of this study was to evaluate the impact of a virtual group-based ACT intervention on intern nurses’ psychological flexibility and social adjustment. As well as the secondary outcome was to assess the impact of an intervention on intern nurses’ work-family conflict.

### Research hypothesizes

- Intern nurses engaged in a virtual group-based ACT intervention can exhibit a higher level of social adjustment than the control group.
- Intern nurses engaged in a virtual group-based ACT intervention can exhibit lower family-work conflict than the control group.
- Intern nurses engaged in a virtual group-based ACT intervention can exhibit less work-family conflict than the control group.

### Research design

This study followed the Consolidated Standards of Reporting Trials (CONSORT) guidelines, utilizing a randomized controlled trial (RCT) design. This RCT was conducted between the beginning of September 2022 and the middle of January 2023, following rigorous guidelines to ensure the validity and reliability of the study’s results. The study employed a two-arm, single-blind approach, including pre-test, post-test, and follow-up measurements, with a waiting list of control groups. The study was registered under the reference number NCT05721339.

### Setting and participants

The study utilized a stratified random sampling method to select participants in proportion to the total number of intern nurses. The four hospitals included in the study were considered separate groups, or strata, and had 806 intern nurses working there during the academic year 2022/2023. These hospitals were the Main University Hospital (El-Miri) (A), with 350 intern nurses; University Hospital Smouha (B), with 200 intern nurses; El Shatby Hospital for Obstetricians and Gynecologists (C), with 106 intern nurses; and El Hadara Orthopedic and Traumatology University Hospital (D) with 150 intern nurses. To be eligible, participants needed beginner intern nurses, aged not more than 25 years, willing to participate, and had no mental health disorders or ongoing psychotherapy.

### Sample size calculation and sampling technique

The sample size was calculated using the G*Power Windows 3.1.9.7 program, with a power of 0.88, an effect size of 0.25, and an alpha error probability of 0.05. Based on the calculation, This study required an a priori sample size of 81 intern nurses randomly selected from the four hospitals using Research Randomizer version 4.0 after considering a 20% loss ratio of follow-up (Hospital A = 35, hospital B = 20, hospital C = 11, hospital D = 15 intern nurses). Using a computer-generated randomization list, the participants were randomly assigned to either the intervention or control group in a 1:1 allocation ratio. The researchers concealed the allocation sequence until the participants were assigned to their respective groups. An independent statistician (one of the study authors) performed all randomization procedures and was blinded to the other authors until intervention procedures were completed. The statistician tried to match the control group as accurately as possible regarding age, gender, and working hour duration.

### Flow diagram

The recruitment process and data collection for a study involving intern nurses. Initially, 90 intern nurses were screened for eligibility to participate in the study. Out of these, 9 refused to participate, and 81 intern nurses were randomly assigned by using Research Randomizer version 4.0 to join either the intervention or control group (*n* = 81) in a 1:1 allocation ratio. The study group consisted of 42 intern nurses, while the control group consisted of 39 intern nurses. All participants in both groups completed the pre-test of the trial (T0) for the study tools, and the study group started their virtual ACT-based intervention. However, 7 intern nurses from the study group attended only two sessions. The remaining 35 participants from the study group completed all 6 sessions of the virtual ACT-based intervention. Also, 4 intern nurses withdrew from the control group during the intervention, so the control group became 35 intern nurses.

**Fig. 2:**
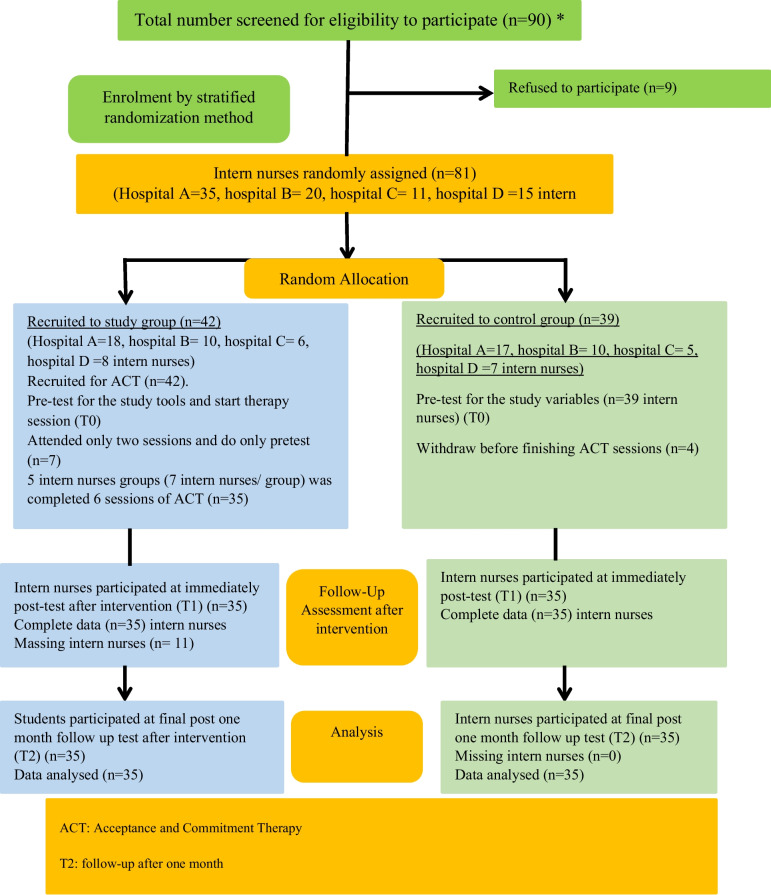
CONSORT

All 70 participants from the study and control groups provided complete data. At post-test measures (T1), the 35 intern nurses from the study group who completed the full virtual ACT-based intervention and the 35 from the control group participated in an immediate post-test after the intervention. At T2, a one-month follow-up test, all 70 participants from the study and control groups participated in a final follow-up test. All 35 participants from the study and control groups provided complete data, and all the collected data were analyzed. The study had a high retention rate, with only 11 intern nurses dropping out. The high retention rate and complete data from all participants provide a reasonable basis for analyzing the effectiveness of the virtual-based ACT intervention for reducing work-family conflicts and improving social adjustment and psychological flexibility among intern nurses. It’s worth noting that for all the dropped 11 intern nurses who could not complete all ACT sessions or withdrew, the researchers emailed electronic copies of the recorded modules and PDF materials (see Fig. 2).

### Procedure

#### Administrative steps

The Faculty of Nursing Alexandria University Research Ethics Committee approved the study protocol. Informed consent was obtained from all participants before they were enrolled in the study. The participants were informed that their participation was voluntary and that they could withdraw from the study without penalty. The participants’ confidentiality was maintained throughout the study. Official written permission was obtained from the Vice Dean of Students’ Affairs Faculty of Nursing in Alexandria, Egypt, to conduct the study. Then the contact information for the intern nurses, including their academic email addresses and phone numbers, was acquired from the University’s Internship affairs unit. Both the intervention and control groups were contacted, and an informational meeting was conducted online with them one week before the commencement of ACT intervention sessions.

#### The control group

The control group received no intervention during the study but was later offered the same web-based ACT intervention. Additionally, an educational program (TAU) for 7 days was delivered through a web-based platform to all intern nurses from all faculty departments, including critical and emergency, medical-surgical nursing, obstetric and pediatric, psychiatric and mental health, and administration. This program covered specific topics that were mandatory for their internship training periods. The program was given to all intern nurses before starting our random allocation to either the intervention or the control group.

#### Intervention

The web-based ACT intervention was developed based on the current literature and guidelines for ACT [18, 26, 27]. The intervention consisted of six biweekly sessions, each lasting 60–90 min, and included video lectures, exercises, and homework assignments. The intervention focused on developing psychological flexibility, adapting to various situations, and achieving valued goals while managing difficult thoughts and emotions. The intervention also aimed to improve participants’ social adjustment and reduce work-family conflict.

#### Implementation phase

The study instruments were converted to electronic formats, and a pilot study was conducted with twenty intern nurses to ensure the research instruments were clear, understandable, and relevant. The pilot study confirmed their clarity, understandability, and relevance. Before the web-based ACT intervention sessions commenced, an online questionnaire was provided to the intervention and control group intern nurses, which they were instructed to complete. To establish rapport, clarify the purpose of the study and the therapy used, and obtain electronic, written consent, each participant was contacted individually for a private online discussion that lasted 15 to 20 min. This communication was conducted through a secure platform. After their consent, the intern nurses in the intervention group were assigned randomly to one of the five smaller groups, each with seven participants undergoing ACT intervention. Afterward, the selected intern nurses were added to a secure and confidential channel on the online platform.

ACT intervention was delivered through a secure online platform with six modules, each with broad goals and specific objectives, led by trained researchers. The modules covered various topics, including mindfulness, values identification, committed action, acceptance, cognitive defusion, and self-as-context. The session’s significant goals and content were translated into Arabic for each session. Each session lasted approximately 90 min, and the entire intervention lasted 3 weeks. During the intervention, the participants were encouraged to complete homework assignments, and weekly reminders were provided. The program sessions included interactive elements to increase participant motivation and enthusiasm, such as worksheets, evaluations with customized feedback, expandable text/popup features, and multimedia, such as photographs, audio recordings, and videos for experiential tasks. To ensure that the participants clearly understood the therapy’s key concepts, each session was led by one researcher, who spent around 60 min explaining the concepts. In contrast, the other researcher served as a mediator and spent about 30 min setting up homework assignments with the participants and answering any questions they might have (see Table 1).

**Table 1:** ACT sessions

**Acceptance and Commitment Therapy (ACT)**		
**Acceptance and commitment therapy (ACT) sessions include the following general outcomes: Participants will be able to do the following:** **▪ Accepting their thoughts and feelings without judging or ignoring them and letting go of the internal conflict with the negative thoughts they have encountered** **▪ Developing patterns of valued committed acts connected to social adjustment and family-work conflict resolution**		
**Session**	**Specific objectives**	**Content and processes**
**Session (1) General outcomes:** **▪ Increase acceptance and willingness** **▪ Diminish experiential control**	▪ Investigate their internal conflict with family members and the workplace in depth to uncover several explanatory types of psychological inflexibility ▪ Apply the psychological inflexibility paradigm to impractical responses to negative experiences ▪ Explore the coping strategies of avoiding and controlling thoughts and feelings prompted by family-work conflict ▪ Identify the negative effect of the controlling agenda ▪ Controlling negative thoughts is the problem, not the solution ▪ As a strategy to coping with the conflict, letting go of the fight with the traditional control agenda ▪ Considering experiencing willingness as an alternative to experiential control	▪ The researchers greeted the participants through online meeting and ask them to open the camera ▪ Inform the participants about the program’s aims and content and establish the therapeutic norms ▪ The group leader displays the ACT components that contribute to psychological flexibility ▪ Encourage students to establish the connection between family-work conflict and psychological inflexibility features ▪ The group leader inspired the students to investigate his typical emotional control agenda as a reaction to conflict and its impact on his life ▪ Confront the students with the typical emotional control agenda that was utilised in reaction to the conflict ▪ The group leader utilised the choice point model to communicate that meaning and to emphasise on acceptance as a facilitator for effective conflict resolution ▪ Introduce the concept of creative hopelessness into the typical emotional regulation agenda ▪ The researcher encourages the students to let go of the fight with conflict and utilise willingness as an alternative, which was demonstrated by practising tug-of-war with the monster metaphor and presenting a film for the terrible guest metaphor ▪ After the class, the homework was to do willingness and acceptance exercises with monster pictures utilising the tug of war was demonstrated by the group mediator
**Session (2) General outcomes:** **▪ Connect with the current moment**	▪ Trying to be aware of what is going on in the outside world to be more resilient, and fully engage in whatever they are doing ▪ Without judgment, observe and describe what appears internally, such as thoughts, feelings, and physical sensations ▪ Observe and identify sources of distraction and avoidance that are employed as a response to work-family conflict that led to be away from the here and now	▪ Integrating their mindfulness abilities, the group leader assisted the students in contacting the present moment to acquire psychological flexibility to deal with the internal conflict ▪ The group mediator was working with the students on the dropping anchor activity ▪ The researcher suggested the students to use observing phrase, such as “I’m observing that…” when referring to behaviour that altered because of internal conflict experiences or any thoughts or emotions expressed throughout the session ▪ The group leader used a film to demonstrate the metaphor river of thoughts to have the students notice the conflicting thoughts and feelings arrive and leave from an observer perspective (mindfulness exercises) ▪ The group mediator assigned the students a homework exercise on a paper worksheet regarding the metaphor of the river of thoughts
**Session (3) General outcomes:** **▪ Diminish cognitive fusion sources** **▪ Accept conflict by distracting yourself from negative ideas**	▪ Recognize and be aware of the causes of cognitive fusion caused by the conflict ▪ connect between fusion causes generated by conflict and impractical activities that cause them to abandon desired life tasks ▪ Analyse and observe the content of negative thoughts for what it really is: pieces of language or words ▪ Create a distance between the negative thoughts and the students	▪ The group leader altered the choice point model to demonstrate the relationship between conflict-induced sources of fusion and their impact on making away moves from the life that the student desires ▪ The leader trained the students to create a separation between the content of negative thoughts and themselves to reduce their influence on behaviour by practising distancing exercises, naming students’ minds exercises, taking your mind for a Walk”, “milk milk milk exercises”, “selling your conflict and thoughts exercises”, and “I am having the thoughts that**…**” ▪ As a homework assignment, the group mediator requests that the students repeat all distance exercises “I am having the idea that……” and “naming your mind.”
**Session (4) General outcomes:** **▪ Differentiate between the conceptualised self and the self as a context**	▪ Monitor and clarify your conflicting experiences, including associated thoughts, feelings, and drives that are likely to alter with time and circumstance ▪ Encourage students to create contact with a sense of self that is continuous, safe, and constant, and from which they may witness and tolerate conflict ▪ Reinforce the idea that the students were not their provoked thoughts and feelings, but that these occurrences varied continually and were a minor element of their personality. As a result, the students performed more effectively, guided by the values they selected	▪ The group leader assisted the students in understanding the many aspects of self-conceptualization by just observing conflict and negative thoughts on various occasions ▪ The leader called the students’ attention to the contrast between the self that observes the negative thoughts or conflict that occur and the self that is the source of those thoughts or conflict ▪ The leader instructed the students to imagine their thoughts, feelings, and conflict content as leaves floating down a stream, and to just observe them as they drifted without attempting to stop or control them. The leaves on the stream metaphor utilised this by displaying a video ▪ The group leader highlighted and emphasised the contrast between self-evaluation products and contents vs the self that evaluates. The difference between ideas and self was demonstrated using the chessboard metaphor video ▪ Students completed homework: worksheet paper for the leaves on the stream metaphor to support the notions of self as context and self as content
**Session (5) General outcomes:** **▪ Determine the value of directions**	▪ Determine their value domains that give their life purpose ▪ Differentiate between value and goal ▪ Define and select goals that match with the values you have chosen ▪ Identify challenges to implementing valued-based living and investigate solutions to overcome those obstacles	▪ The group facilitator addressed with the students about how previous attempts at handling conflict had a negative impact on and interfered with their ability to achieve their objectives, which in turn had a negative impact on their values ▪ The group leader guided the participants as they investigated potential moral values that may give their life purpose ▪ The leader utilised a bull’s eye worksheet to assist the students in identifying and classifying their values to explain why they were consciously abandoning their conflict-avoidance, control-coping strategies, and integrating ACT into their life ▪ Using the metaphor of a compass, the leader helped the students understand the distinction between value and goal and how value relates to objectives ▪ Using a worksheet based on the selected value from the bull’s eye, the group mediator assisted the students in setting SMART goals and taking actions based on those goals ▪ Students were assigned homework that contained a goal that was congruent with one of the values they had picked from the bull’s eye worksheet
**Session (6) General outcomes:** **▪ Create and refine a pattern of committed behaviour**	▪ Specify and establish objectives that are in line with their values ▪ Address the barriers preventing target achievement ▪ Despite the existence of unpleasant conflict and associated thoughts and feelings, resolve to pursue a worthwhile life goal ▪ To get through such obstacles, make use of all six ACT model fundamental processes	▪ The group facilitator concentrated on utilising the bully’s eye to identify values ▪ The researcher has selected one of the bully’s value areas and is beginning to develop an action plan for it ▪ The students created a list of 10 distinct tasks that relate to their short-term objectives and are compatible with the selected value. They then graded the tasks according to how challenging they are to complete ▪ The group leader kept track of the obstacles and challenges that would prevent them from attaining their objectives, as well as how they overcame them when they occurred ▪ To persuade the students to be willing to stick with the action plans despite obstacles, the group mediator demonstrated the Passengers-on-the-Plan Metaphor in a video and worksheet ▪ The students’ opinions on the ACT sessions were gathered

#### Session time plan

**Table:** 

Session time plan (90 min)	**• 5 min:** practicing mindfulness, noticing self, and observing g external environment • 15 min: revision for the previous concepts and obstacles that face the intern nurses during skills demonstration • 30 min: applying the new concepts of the session and demonstrating them through videos and live stream simulation by the trained researcher • 30 min: Discussed with the intern nurses the session’s aim and skills and made them re-demonstrate the techniques they learned during the session • 10 min: take-home massages and assigned homework

After each session, participants were given a brief therapeutic homework assignment and access to a recorded module. Follow-up letters included a URL to the next session and download links for worksheets or multimedia from the prior session.

#### Follow up

To assess the efficacy of the therapy, a post-assessment survey was conducted using the Work-related Acceptance and Action Questionnaire, the Social Adjustment Scale-Self Report (SAS-SR), and the Work-Family Conflict Scale (WFCS) on both the study (*n* = 35) and control groups (*n* = 35) within 4 to 7 days after completing the ACT sessions. A post-one-month test was also conducted using the same tools on the study group (*n* = 35) and control group (*n* = 35) to assess the efficacy and sustainability of therapy concepts.

#### Data collection

Data were collected at baseline (T0), post-intervention (T1), and follow-up (T2) using online questionnaires. The data collection tools included the sociodemographic data, the Work-related Acceptance and Action Questionnaire, the Social Adjustment Scale-Self Report (SAS-SR), and the Work-Family Conflict Scale (WFCS). The questionnaires were administered through an online survey platform, and the participants were given a unique identification number to maintain their anonymity.

### Data collection measures

#### Intern-nurses’ sociodemographic data profile

The survey designed by the researchers consisted of questions regarding the age, gender, place of residence, weekly working hours, and income of the intern nurses. Additionally, the survey included inquiries about the participants’ mental and physical health status and whether they were currently undergoing any type of psychotherapy.

### Primary outcome measures

#### Work-related Acceptance and Action Questionnaire (WAAQ) (English version)

Bond et al. [26] developed the Work-related Acceptance and Action Questionnaire (WAAQ) to assess psychological flexibility in professional contexts. It consists of seven statements assessed on a Likert Scale from 1 (Never true) to 7 (Always true), such as “I can admit my faults at work and yet be successful” and “I can work efficiently even when I doubt myself”. The items stand for people’s ability to take goal-directed action in the face of unpleasant internal experiences. WAAQ scores range from 7 to 49, with higher scores suggesting greater psychological flexibility. The WAAQ has an elevated level of internal consistency (Cronbach’s alpha of 0.85) and test–retest reliability (ICC of 0.85) [28]. In this study, Cronbach’s alpha coefficient of the scale total in the current study was 0.84.

#### Social Adjustment Scale—Self Report (SAS-SR) (English version)

The SAS-SR is a popular 54-item self-reported social adjustment scale that assesses instrumental and expressive role performance over the previous two weeks [29]. It addresses six areas of functioning: employment (as a paid worker, unpaid homemaker, or student), social and leisure activities, interactions with extended family, marital partner role (if relevant), parenting role (if applicable), and role within the family unit (including perceptions of economic functioning). The elements in each of the six categories encompass four sorts of content: performance at anticipated tasks, interpersonal friction, finer characteristics of interpersonal relationships, and emotions and satisfactions. The full-length SAS-SR takes around 15–20 min to administer. The researchers employed three sections in this study: the working role, social and leisure time activities, and connections with extended family. The higher the scores, the more impaired the person is (i.e., lower levels of social adjustment). The SAS-SR provided a satisfactory level of internal consistency, with a mean Cronbach’s coefficient of 0.74. Appropriate test–retest correlations were also observed, with a correlation of *r* = 0.80 [30]. In this experiment, Cronbach’s alpha coefficient of the scale total was 0.94.

### Secondary outcome measures

#### Work-Family Conflict Scale (WFCS) (English version)

The WFCS was constructed by Netemeyer et al. [31]. Work-family conflict (WFC) and family-work conflict (FWC) were divided on the scale (FWC). Each subscale comprised five items that measured the level of conflict in that subscale. It is evaluated on a 7-point Likert scale, with one being “very strongly disagree” and seven being “very strongly agree”. The items for each subscale are totaled to supply the overall scores of WFC (ranging from 7 to 35) and FWC (ranging from 7 to 35); higher scores show higher degrees of conflict. Cronbach’s alpha was 0.88 overall [32]. In the current study, Cronbach’s alpha coefficient for the scale was 0.75.

### Data analysis

The data were analyzed using the Statistical Package for Social Sciences (SPSS) version 26.0. Descriptive statistics were used to summarize the demographic characteristics of the participants. The normality of the distribution of quantitative data was assessed using the Kolmogorov–Smirnov test. The independent samples t-test was used to compare the mean scores of the two groups at baseline. The within-group and between-group differences in the T0, T1, and T2 outcome measures were analyzed using repeated-measures ANOVA. One-tailed tests were used to determine statistical significance, with the significance level at *p* < 0.05.

## Results

Table 2 presents the distribution of sociodemographic characteristics for two groups of intern nurses, a study group (*n* = 35) and a control group (*n* = 35). In the study group, 25.7% of participants were male, while in the control group, 31.4% were male. The difference was not statistically significant (χ2 = 0.280, *p* = 0.597). Regarding age, the mean age for the study group was 22.27 years (SD = 1.55), while for the control group, the mean age was 22.0 years (SD = 1.12). The difference was not statistically significant (*t* = 0.696, *p* = 0.488). Most participants in both groups were single, with 94.28% in the study group and 97.14% in the control group. The difference was not statistically significant (χ2 = 0.022, *p* = 0.881). Concerning place of residence, most participants in both groups lived in urban areas, with 74.0% in the study group and 73.5% in the control group. The difference was not statistically significant (χ2 = 0.001, *p* = 0.971). Most participants in both groups had 2–5 family members, with 65.7% in the study group and 62.9% in the control group, while the rest had 5–10 family members. The difference was not statistically significant (χ2 = 0.062, *p* = 0.803). Also, the previous practical nursing experience showed that 34.3% of participants in the study group and 51.4% in the control group reported having previous practical nursing experience. The difference was not statistically significant (χ2 = 2.100, *p* = 0.147). Working during an internship revealed that 31.4% of participants in the study group and 34.3% in the control group reported working during internship training. The difference was not statistically significant (χ2 = 0.065, *p* = 0.799). The mean working hours for both groups were 56 h per week, with a standard deviation of 8 h for the study group and 6 h for the control group. The difference was not statistically significant (*t* = 0.408, *p* = 0.703). This table demonstrates no statistically significant difference in the sociodemographic characteristics between the study and control groups, proving that the two groups are equivalent.

**Table 2:** Distribution of the studied intern nurses according to their socio-demographic characteristics

**Socio-demographic characteristics**	**Study group (*n* = 35)**		**Control group (*n* = 35)**		***Test***	***P***
	***N***	***%***	***N***	***%***		
**Gender**						
**Male**	9	25.7	11	31.4	***χ***^***2***^ = 0.280	0.597
**Female**	26	74.3	24	68.6		
**Age**						
**Mean (SD)**	22.27 (1.55)		22.0 (1.12)		t = 0.696	0.488
**Marital status**						
**Single**	33	94.28	34	97.14	***χ***^***2***^ = 0.022	0.881
**Married**	2	5.72	1	2.86		
**Residence**						
**Urban**	25	74.0	24	73.5	***χ***^***2***^ = 0.001	0.971
**Rural**	10	26.0	11	26.5		
**Number of Family members**						
**2–5**	23	65.7	22	62.9	***χ***^***2***^ = 0.062	0.803
**5–10**	12	34.3	13	37.1		
**Did you have any practical nursing experiences before internship training?**						
**Yes**	12	34.3	18	51.4	***χ***^***2***^ = 2.100	0.147
**No**	23	65.7	17	48.6		
**Do you work during your internship training?**						
**Yes**	11	31.4	12	34.3	***χ***^***2***^ = 0.065	0.799
**No**	24	68.6	23	65.7		
**Do you complain from any physical illness?**						
**Yes**	12	34.3	7	20	***χ***^***2***^ = 1.806	0.179
**No**	23	65.7	28	80		
**Financial income**						
**Sufficient**	19	54.3	15	42.9	***χ***^***2***^ = 1.271	0.530
**Sufficient to some extent**	14	40.0	16	45.7		
**Insufficient**	2	5.7	4	11.4		
**Working hours**						
**Mean (SD)**	56 (8)		56 (6)		t = 0.408	0.703

X^2^: Pearson Chi-square test, t: Independent t-test

^*^Statistically significant *p*-value at ≤ .05

Table 3 illustrates the mean scores and standard deviations of the Work-related Acceptance and Action Questionnaire (WAAQ) for the study and control groups pre-intervention, immediate post-intervention, and one-month post-intervention. The higher mean scores indicate greater psychological flexibility. The results show that at the pre-intervention stage, the mean score of the study group (31.00) was significantly lower than the control group (34.62), with a statistically significant difference (*p* = 0.036). This suggests that the two groups were not initially equivalent regarding psychological flexibility. However, immediately after the intervention, the mean score of the study group (43.11) was significantly higher than that of the control group (34.15) with a statistically significant difference (*p* < 0.001), indicating a significant improvement in psychological flexibility in the study group compared to the control group. One month after the intervention, the mean score of the study group (41.88) remained significantly higher than that of the control group (33.21), with a statistically significant difference (*p* < 0.001), indicating that the effect of the intervention was sustained over time. The ANOVA results indicate a statistically significant difference between the study and control groups regarding WAAQ scores across all three stages (F = 128.457, *p* < 0.001, η2 = 0.791), suggesting a large effect size.

**Table 3:** The mean scores and standard deviations of Work-related Acceptance and Action Questionnaire (WAAQ) for the study and control groups at pre, immediately post, and post one month of the intervention

**WAAQ**	**Study group (*n* = 35)**		**Control group (*n* = 35)**		**t (p)**
	***M***	***SD***	***M***	***SD***	
**Pre-intervention**	31.00	6.30	34.62	7.53	-2.142 (.036)^*^
**Immediately post**	43.11	3.15	34.15	6.96	6.681 (.000)^**^
**Post 1 month**	41.88	3.89	33.21	5.46	7.526 (.000)^**^
**F**	128.457		5.762		
**(P)**	.000^**^		.005^*^		
**η**^**2**^	.791		.157		

The higher mean suggesting greater psychological flexibility

t = independent t- test, F = ANONA, η^2^ = Partial Eta Squire

*WAAQ* Work-related Acceptance and Action Questionnaire

^*^Statistically significant *p*-value at ≤ .05

^**^Statistically significant *p*-value at ≤ .001

Table 4 presents the mean scores and standard deviations of the Social Adjustment Scale—Self Report (SAS-SR) for the study and control groups pre-intervention, immediate post-intervention, and one-month post-intervention. The lower mean scores indicate higher levels of social adjustment. The results show that at the pre-intervention stage, the study group had significantly lower mean scores than the control group in all four subscales of the SAS-SR (working role, spare time activities, role within the family unit, and total score), with statistically significant differences (*p* < 0.001) and large effect sizes (η2 = 0.932). This suggests that the two groups were not initially equivalent regarding social adjustment. Immediately after the intervention, the mean scores of the study group improved significantly in all four subscales of the SAS-SR, with statistically significant differences (*p* < 0.001) and large effect sizes (η2 = 0.906).

**Table 4:** Description of mean scores and standard deviations of Social Adjustment Scale—Self Report (SAS-SR) for the study and control groups at pre, immediately post, and post one- month of the intervention

***SAS-SR***	***Study group (n* = *35)***		***Control group(n* = *35)***		***T (p)***
	***M***	***SD***	***M***	***SD***	
**Working Role Subscal**					
**Pre**	18.48	3.83	12.50	1.58	8.470 (0.000)^**^
**Immediately**	9.74	2.13	12.78	1.82	-6.277 (0.000)^**^
**Post**	13.37	3.83	15.03	2.91	-2.291 (0.025)^**^
**F**	32.478^**^		36.435^**^		
**(P)**	.000		.000		
**η**^**2**^	.489		.540		
**Spare Time Activities Subscale**					
**Pre**	36.42	5.04	18.28	2.78	18.420 (0.000)^**^
**Immediately**	22.88	3.53	18.62	2.61	5.565 (0.000)^**^
**Post**	22.31	4.19	25.43	4.89	-2.809 (0.007)^**^
**F**	327.659^**^		84.289^**^		
**(P)**	.000		.000		
**η**^**2**^	.906		.731		
**Role within the family unit**					
**Pre**	21.77	4.54	11.28	1.83	12.580 (0.000)^**^
**Immediately**	12.20	2.98	11.65	1.73	.900 (0.372)
**Post**	13.20	2.68	15.59	3.04	-3.417 (0.001)^**^
**F**	318.183^**^		72.854^**^		
**(P)**	.000		.000		
**η**^**2**^	.903		.702		
**Total Score**					
**Pre**	76.68	10.86	42.06	4.64	17.205 (0.000)^**^
**Immediately**	44.82	6.30	43.06	4.33	1.324 (0.190)
**Post**	48.77	7.64	56.06	8.92	-3.599 (0.001)^**^
**F**	468.051^**^		103.913^**^		
**(P)**	.000		.000		
**η**^**2**^	.932		.770		

The lower mean scores indicate higher levels of social adjustment

t = independent t- test, F = ANONA, η^2^ = Partial Eta Squire

*SAS-SR* Social Adjustment Scale—Self Report

^**^statistically significant *p*-value at ≤ .001

The ANOVA results indicate significant differences between the study and control groups regarding SAS-SR scores across all subscales and stages, with large effect sizes. In contrast, the control group showed no significant changes in mean scores. One month after the intervention, the mean scores of the study group remained significantly higher than those of the control group in the Working Role and Total Score subscales, with statistically significant differences (*p* = 0.025 and *p* < 0.001, respectively) and moderate effect sizes (η2 = 0.157 and 0.770, respectively). However, the two groups had no significant differences in the Spare Time Activities and Roles within the Family Unit subscales.

Table 5 presents the mean scores and standard deviations of the Work-Family Conflict Scale (WFCS) for the study and control groups pre-intervention, immediate post-intervention, and one-month post-intervention. The higher mean scores indicate higher degrees of conflict. The results show that at the pre-intervention stage, the study group had significantly higher mean scores than the control group in all three subscales of the WFCS (Work Interference with Family, Family Interference with Work, and Total Score), with statistically significant differences (*p* < 0.001) and large effect sizes (η2 = 0.851). Immediately after the intervention, the mean scores of the study group improved significantly in all three subscales of the WFCS, with statistically significant differences (*p* < 0.001) and large effect sizes (η2 = 0.911). This suggests that the two groups were not initially equivalent regarding work-family conflict.

**Table 5:** Description of mean scores and standard deviations of Work-Family Conflict Scale (WFCS) for the study and control groups at pre, immediately post, and one-month post the intervention

**WFCS**	***Study group (n* = *35)***		***Control group (n* = *35)***		***T (p)***
	***M***	***SD***	***M***	***SD***	
**WFCS**					
**Pre**	25.42	5.33	19.65	5.73	4.270 (.000)^**^
**Immediately**	15.31	3.95	21.21	5.96	-4.813(.000)^**^
**Post**	15.97	3.56	23.62	3.87	-8.417(.000)^**^
**F**	194.107		36.795		
**(P)**	.000^**^		.000^**^		
**η**^**2**^	.851		.543		
**FWCS**					
**Pre**	22.00	3.81	18.46	2.68	4.411(.000)^**^
**Immediately**	8.97	2.92	20.68	3.44	-15.034(.000)^**^
**Post**	13.00	3.40	23.62	3.25	-13.039 (.000)^**^
**F**	346.853		111.985		
**(P)**	.000^**^		.000^**^		
**η**^**2**^	.911		.783		
**Total Score**					
**Pre**	47.42	7.22	38.12	7.25	5.253(.000)^**^
**Immediately**	24.28	5.71	41.90	8.34	-10.161(.000)^**^
**Post**	28.97	5.33	47.25	5.80	-13.371(.000)^**^
**F**	567.188		135.962		
**(P)**	.000^**^		.000^**^		
**η**^**2**^	.943		.814		

The higher mean reflects higher degrees of conflict

t = independent t- test, F = ANONA, η^2^ = Partial Eta Squire

*WFCS* Work-Family Conflict Scale

^**^statistically significant *p*-value at ≤ .001

In contrast, the control group showed no significant changes in mean scores. One month after the intervention, the mean scores of the study group remained significantly lower than those of the control group in all three subscales of the WFCS, with statistically significant differences (*p* < 0.001) and large effect sizes (η2 = 0.943). The ANOVA results indicate significant differences between the study and control groups regarding WFCS scores across all subscales and stages, with large effect sizes.

## Discussion

Work and family are the two most influential aspects of a person’s life. The balance between a person’s work and family life has drawn more and more research attention. The conflict between work and family can adversely affect an organization’s and an individual’s health and well-being [8]. Research is necessary to describe, understand, and address work-family conflict because it arises from how people perceive it [19]. According to researchers, studies on social adjustment for healthcare staff and nursing interns are scarce in Egypt. Recently, ACT has been utilized to treat various psychological issues and challenges at work [33]. Therefore, the present study aimed to evaluate the impact of virtual group-based ACT on intern nurses’ social adjustment and work-family conflict.

The researchers in the present study used the work-related acceptance and action questionnaire to measure the effect of ACT on improving work-related psychological flexibility among intern nurses. The current findings reflect a statistically significant difference between the study and control group concerning work-related psychological flexibility after finishing the intervention and after a one-month follow-up with a large effect size of 79%. It can be said that ACT trains students to maintain their attention on the present and take appropriate action toward achieving their goals and upholding their values, even when faced with challenging or unwanted psychological events such as thoughts, feelings, physiological sensations, images, and unwanted memories.

Moran [34] explained that acceptance skills teach employees to behave effectively even during stressful emotions and sensations, increasing psychological flexibility. ACT training teaches people that feelings, urges, memories, and other private psychological experiences occur naturally throughout the workday and should not be eliminated or avoided. This approach implies that contacting psychological experiences willingly is healthier and thus makes the worker more effective.

The current study’s findings revealed a substantial difference in intern nurses’ work-family conflict ratings between the study and control groups. In other words, ACT reduced work-family conflict in the study group compared to the control group before and after, and the effect was sustained with a large effect size (94.3%) throughout a one-month follow-up. However, most intern nurses in the intervention group reported suffering from value conflict between their family and work. In line with our findings, Sabil et al. [20] reported that nurses had varying degrees of conflict while attempting to meet work expectations and family roles. Similarly, Guille et al. [35] surveyed 3121 interns from 44 medical schools in the United States during the 2015–2016 academic year. They found that both males and females experienced an increase in work-family conflict and depression symptoms during the first six months of the internship year, and there was a correlation between the two. Sedoughi et al. [36] explained that nurses spent more time at work than with their families and were more satisfied with their family lives than their profession. Leineweber et al. [37] revealed that the conflict between work and family life affects both men’s and women’s health, even though women experience it significantly more frequently than men. In a study of five Italian public hospitals conducted by Zurlo, Vallone, & Smith [6], 450 nurses (206 males, 244 females) took part. They found differences between the sexes in perceived levels of WFC, anxiety, depression, and somatization with nurses performing. Still, there were no differences in perceived job control, social support, or job satisfaction.

The researchers focused on the two essential areas of life rather than on two conflicting values. They used different ACT skills, like acceptance and present-moment exercises, to overcome thoughts and feelings that interfered with the chosen values. As well as the researchers used the tug-of-war with a monster metaphor as an example to increase the intern nurses’ awareness about accepting their conflicting thoughts rather than resisting them. A group of intern nurses was asked to imagine pulling the robe with a researcher, giving them a chance to provide alternative solutions to that battle when the monster on the other side of the robe was unbeatable. They were convinced to accept and let go of the struggle with their perplexing thoughts and feelings rather than suppress them or avoid them experientially.

The present study finding is congruent with a study entitled “Comparison between the effects of group-based ACT and group-based reality therapy on work-family conflict & psychological well-being of married female staff,”. A study using a work conflict questionnaire on 45 employees found that group-based ACT can be utilized to increase psychological well-being and group-based reality therapy can be an effective method for reducing work-family conflicts [38].

ACT improved social adjustment compared to the control group before and after, and the effect persisted over a one-month follow-up. The improvement in social adjustment was remarkably observed in all social adjustment subscales in the study group. It can be regarded as a decrement in the mean scores of the working role subscale, spare time activations, and role within the family unit. These results follow Tanhadoost et al. [39] study of the effectiveness of ACT intervention on loneliness and social adjustment among girls with a social anxiety disorder. They confirmed that ACT improves social adjustment.

Mirmoeini et al. [9] investigate how acceptance- and commitment-based treatment affects social anxiety and adjustment in high school males. Similarly, Ostadian et al. [40], in a quasi-experimental study of 30 males with a physical disability (15 control group and 15 study group). They claimed that a group intervention focused on acceptance and commitment improved social adjustment. The experimental group had six training sessions, with one weekly session lasting 90 min. The findings revealed that ACT impacts high school boys’ social anxiety and adjustment. Such improvements might be rationalized by the activation of normalizing and accepting attitudes toward the intern nurses’ complex thoughts and feelings as an inherent component of the ACT skills. The intern nurses in the study practiced recognizing their challenging ideas as just thoughts, accepting them, and adopting behaviors congruent with their intended values. During these challenging circumstances, the intern nurses also learned to master motivation and conduct planned activities. Some trainees indicated that present-moment exercises and mindfulness allowed them to shift their attention away from their confusing thoughts and feelings, allowing them to focus on essential acts in their life that can enhance their sense of self-worth and self-actualization.

To explain the effect of ACT on social adjustment, we can say that today’s world requires more flexibility and individual and social adjustment than ever. Adaptability and flexibility can improve people’s lives [9]. Individuals with high levels of mindfulness are more aware of their body sensations, thoughts, and emotions with less reactivity, and they maintain a stance of equanimity rather than engaging in suppression or excessive fixation. The non-judgmental perspective and acceptance-based approach facilitate understanding and accepting intrapersonal and interpersonal challenges. The clarity and vividness of the experience and orienting to the present moment with curiosity and openness can help one’s psychosocial adjustment to life changes and new environments [41].

According to the current study findings, sustained improvement in work-family conflict, social adjustment, and work-related psychological flexibility after one month of implementing ACT might be explained by strengthening problem-solving abilities as an essential part of the ACT process. They were encouraged to find different methods to deal with their troubling thoughts and feelings. By this time, these attitudes had been acquired and internalized and could be employed autonomously and successfully. Another possible explanation is the effect of working memory consolidation caused by producing alternate solutions in metaphor demonstration.

### Limitations of the study

The study had several limitations. There was no long-term follow-up beyond one month to assess the sustained effect of ACT on work-family conflict, work-related psychological flexibility, and social adjustment. Individual differences, such as personality traits, cultural factors, and prior experiences, were not assessed in relation to the effectiveness of ACT interventions. Lack of control was another limitation in the present study, as intern nurses may not adhere to the intervention as strictly as they would in an in-person setting, which could affect the validity of the results and limit the ability to draw conclusions about the effectiveness of the intervention. Uncontrolled contamination between the study and control groups due to participants meeting each other during their shifts could affect the validity of the results. The presence of baseline differences between the groups indicates that the randomization process may not have been completely successful. As a result, it may be challenging to draw accurate conclusions about the efficacy of the intervention. Excluding participants who did not complete the intervention introduced biases and limited generalizability. Future studies could consider more rigorous randomization procedures and collecting more detailed baseline information to address these limitations.

## Conclusion and recommendations

ACT delivered in a virtual group setting with intern nurses is a viable method for increasing work-related psychological flexibility and social adjustment while decreasing work-family conflict immediately after intervention and sustained for one month after implementation; the study group experienced a significant difference from the control group. As well as further studies were needed with a large sample size to explore the long-term effects of virtual group-based ACT intervention on intern nurses’ social adjustment and work-family conflict. Future studies could investigate the sustainability of the effects of ACT beyond one month and examine the impact of ACT on other outcomes such as burnout, job satisfaction, and turnover intentions. Moreover, it would be interesting to investigate the effectiveness of ACT interventions on other healthcare professionals, such as physicians and allied health professionals, and explore the potential benefits of integrating ACT into healthcare training programs. Additionally, future research could investigate the effectiveness of different delivery modes, such as individual versus group-based ACT interventions, and examine the feasibility of implementing ACT in different cultural contexts.

### Implication for nursing management

The findings of this study suggest that academic and hospital administrators pay attention to the importance of social maladjustment and the impact of workplace conflict on intern nurses. Group-based ACT intervention can help intern nurses develop a different mindset, identify their limitations, and overcome them with the support of others. So, they tend to participate more in social communication, which fosters positive thinking in the person. The results of the present study propose that Group-based ACT intervention as a transdiagnostic program must be involved in the preparatory courses and interventions among intern nurses to increase work-related psychological flexibility and decrease work-family conflicts.

## Data Availability

The datasets used and/or analyzed during the current study are available from the corresponding author upon reasonable request.

## Abbreviations

ACT: Acceptance and commitment therapy
WFC: Work-family conflict
RFT: Relational frame theory
WAAQ: Work-related acceptance and action questionnaire
SAS-SR: Social adjustment scale- self-report
WFCS: Work-family conflict scale
CONSORT: Consolidated Standards of Reporting Trials
RCT: Randomized Control Trial
TAU: Treatment as Usual
